# Wilson’s disease and diagnostic conundrum in a low income country

**DOI:** 10.11604/pamj.2017.26.201.11377

**Published:** 2017-04-13

**Authors:** Pratap Kumar Patra

**Affiliations:** 1 Department of Pediatrics, ALL India Institute of Medical Sciences, Patna, Bihar, India

**Keywords:** Wilsons disease, diagnostic dilemma, resource limited setting

## Abstract

Wilson’s disease is a well-known leading cause of chronic liver disease in children. However it may remain undiagnosed in a resource limited setting for a long period. We describe a six year male child diagnosed Wilson’s disease with extreme elevation of liver enzymes which is not reported earlier. The diagnosis was also baffling because of inconsistency of other laboratory parameters.

## Introduction

Wilson disease is one of the common metabolic liver disorders prevailing worldwide. The diagnosis is always difficult in children [1]. It is also a chronic liver disease for which effective therapy is available therefore, early diagnosis is crucial. However because of its varied range of clinical manifestation, at times the diagnosis is clinically challenging. Early diagnosis is essential to prevent progression of the underlying disease condition and to screen other siblings. Though various laboratory investigations are there to support the diagnosis, there are many pitfalls associated with it, thereby it always pose a diagnostic dilemma even in tertiary care institutes. The diagnosis is further complicated when investigations does not support the condition.

## Patient and observation

A six year male child presented with chief complaints of yellow discoloration of eyes and associated history of passing high coloured urine for three months. It was associated with nausea, diffuse abdominal pain and decreased appetite. There was no history of irritability, change in behaviour or altered sleep pattern and no bleeding from any site. He had not received blood transfusion any time in the past. There was no similar illness any of the family members. The child was admitted to a private institution for a two week period, but as there was no improvement, he was referred to our institute. At admission, he was afebrile and vitals were stable. General physical examination revealed mild pallor and severe jaundice. Gastrointestinal systemic examination revealed moderate hepatomegaly along with an enlarged left lobe of liver. There were no other clinical features suggestive of chronic liver disease. Investigations revealed a total serum bilirubin of 15.6mg/dl with conjugated fraction of 13.40mg/dl. Serum glutamate pyruvate transaminase (SGPT) level was 1059/IU and serum glutamate oxaloacetate transaminase (SGOT) 2239/IU. Alkaline transferase level was 290 IU. Prothrombin time was 17 second. Viral markers for Hepatitis B surface antigen (HbsAg), Hbe Ag, Anti HCV, Anti HAV IgM, Anti HEVIgM were negative. Antinuclear antibody was also negative. Serum albumin and globulin was 3gm/dl and 4gm/dl respectively. Hemogram revealed haemoglobin of 11.5gm%, total leukocyte count 7,400 with differential count of N35, L55, E08, and M02. Serum electrolytes and urea, creatinine was within normal limit. Slit lamp examination of the eyes was normal. Serum ceruloplasmin level was 48mg/dl and 24 hour urinary copper excretion was 217µg/day. However, the 24 hour copper excretion after d-penicillamine challenge was 48µg/24 hours. Parents were not affording for liver biopsy and estimation of copper. Considering provisional diagnosis of Wilsons disease based on 24 hour urinary cupper excretion report and elevation of SGOT more than SGPT we started d-penicillamine along with pyridoxine. After 6 weeks of follow up he was much improved. Repeat serum bilirubin was 1.5mg/dl and SGOT 74 IU and SGPT was 56 IU respectively.

## Discussion

Wilson’s disease one of the common metabolic disorder in developing countries and attributes to 6-21% of chronic liver disease in children [2,3]. Though it’s a common clinical entity in childhood, the diagnosis is clinically challenging. The early recognition of Wilson’s disease is very important by clinical, biochemical or genetic examination so that the progression of the liver disease can be prevented [4]. However, In contrast to developed countries, the ancillary investigations for Wilson’s disease are available only few tertiary care institute in India. The marked elevation of liver enzymes usually suggests hepatic injury, either caused by virus, drugs or toxins and circulatory shock. However, the index case had marked elevation of liver enzymes which is not reported earlier in Wilson’s disease. The ratio of AST to ALT more than 2 is suggested of chronic liver disease either secondary to alcohol or Wilsons disease [5]. In the index, case the marked elevation of AST in comparison to ALT was the early clinical clue towards the underlying condition. The serum ceruloplasmin is low in most of the children with Wilson’s disease but it may be normal 5-40% case [6]. Estimation of serum ceruloplasmin by radial immunodiffusion method gives higher value because it measures the inactive apoceruloplasmin and most of the laboratory uses this method. The standard method is copper oxidase method but it is technically difficult. The 24 hour urinary copper excretion in this case was another indication towards the underlying condition. In untreated symptomatic children baseline copper excretion greater than 100µg/24 hour is diagnostic of Wilson’s disease [7]. However the D-penicillamine challenge did not yield abnormal results in our case. The sensitivity of D-penicillamine test had only sensitivity of 12.5% in comparison to children with other liver disease [8]. Though liver biopsy is thought to be the gold standard; it has its own limitations. It is an invasive procedure and because the deposition of copper in the liver is non-homogenous false negative report is not uncommon, especially in later stages of disease. The other disadvantage is prolongation of prothrombin times which precludes the liver biopsy. In our country, hepatic copper estimation is available only in few centres which is a major barrier in early and accurate diagnosis. The direct molecular genetic study is also difficult because of there are more than 500 possible mutation.

## Conclusion

In the era of evidence base medicine where we are much dependent on the laboratory investigation, laboratory parameters may be perplexing in Wilson’s disease. The extreme elevation of liver enzymes in Wilson’s disease is unusual and has not been reported before. In low income country and resource limited set up clinicians should diligently interpret and use these parameters in support of their patient care.
